# No dislocation and low complication rate for a modern dual mobility cup with pre-impacted femoral head in primary hip replacement: A consecutive series of 175 hips at minimum 5-year follow-up

**DOI:** 10.1051/sicotj/2022050

**Published:** 2023-01-17

**Authors:** Constant Foissey, Cécile Batailler, Vishal Rajput, Aditya B.J. Premkumar, Elvire Servien, Sébastien Lustig

**Affiliations:** 1 Department of Orthopedic Surgery and Sport Medicine, Croix-Rousse Hospital, FIFA Medical Center of Excellence 69004 Lyon France; 2 Université de Lyon, Université Claude Bernard Lyon 1 69100 Lyon France; 3 The Midyorkshire Hospitals NHS Trust WF14DG Wakefield United Kingdom; 4 Fortis Hospital 560076 Bangalore India; 5 EA 7424, Interuniversity Laboratory of Human Movement Science, Université Lyon 1 69100 Lyon France; 6 Université de Lyon, Université Claude Bernard Lyon 1, IFSTTAR, LBMC UMR_T9406 69622 Lyon France

**Keywords:** Total hip arthroplasty, Dual mobility cup, Survival, Dislocation, Complication, Loosening

## Abstract

*Introduction:* Despite its excellent results in preventing dislocation, the dual mobility cup (DMC) is still struggling to be adopted by some teams due to premature wear and loosening reported on first-generation implants. Therefore, this study aimed to assess the mid-term survivorship of a modern DMC with a pre-impacted head and the radio-clinical results at a minimum follow-up of 5 years. *Methods*: This was a retrospective single-centre study performed on patients who had a primary total hip replacement for osteoarthritis in 2016. The cup was a third-generation DMC with a pre-impacted femoral head. Clinical (harris hip score (HHS)) and radiological (cup abduction, anteversion, overhang, and radiolucent lines) results were recorded, as well as complications, particularly dislocations and survivorship. A minimum of five years of follow-ups was required. *Results*: One hundred and seventy-five hips (167 patients) met the inclusion criteria. Five hips (2.9%, 5/175) were lost to follow-up and excluded from the postoperative analysis. The mean follow-up period was 70 ± 2.9 months [63.6–76.5]. Three cups needed revision surgery (1.8%, 3/170): one for septic loosening, and two for chronic infection. At 77 months, the global survival probability was 98.2% ± 1, and the survival probability excluding septic aetiology was 100%. There was a significant improvement in the HHS from pre-operatively (48.3 ± 6.0 [14.0–70.0]) to post-operatively (96 ± 4.5 [50–100]) (*p* < 0.0001). There were no postoperative dislocations recorded, nor any iliopsoas-impingement or symptomatic cam-effect. *Discussion*: This study showed excellent survival and good radiological and clinical results of this dual mobility cup at a mid-term follow-up. None of the patients had dislocation or any specific complication feared with dual mobility cups.

## Introduction

The efficiency of dual mobility cups (DMC) in reducing dislocation is no longer to be proven. Since its introduction in France by Gilles Bousquet in 1974 [1], many studies have shown its efficiency in trauma, primary and revision surgeries [2–7]. These promising results were observed very soon in the original studies using first-generation DMC [8].

Nevertheless, time-specific issues revealed themselves: random long-term biological fixation due to simple alumina coating [9]; too prominent design leading to iliopsoas impingement muscle and the femoral neck [10, 11]; premature wear of the third articulation leading to intra-prosthetic dislocation (IPD) due to the absence of a chamfer, a poor quality conventional polyethylene, and a non-adapted wide and rough femoral neck [12–15]. Work on all of these factors (combined titanium and hydroxyapatite coating, ultra-high molecular weight polyethylene (UHMWPE), chamfers in the third articulation, pre-impacted femoral head and thin polished round neck) led to the introduction of second-generation DMCs in the late 1990s, with improved survival and almost eliminated IPDs [16–18]. Due to improved results, its use is increasingly widespread, especially in France, its native country (39.3% in primary surgery, 87.9% in revision surgery in 2018) [19, 20]. On the other hand, some teams and countries are still hesitant, they reserve it for particular indications and look for more robust results to extend their indications [20, 21].

In order to improve every potential default, the implants continue to evolve. That is why recently, in addition to the improvements mentioned above, a DMC with a pre-impacted head has been developed. This solution, in addition to saving operative time, handling and storage, makes it possible to secure the impaction which is done perfectly in the axis in a robotized way and thus protects the third articulation, in particular, the head capture mechanism which can be damaged if not impacted properly in the operating room [22]. This study aimed to assess the mid-term survivorship of modern dual mobility cups (DMC) with pre-impacted heads, as well as the radio-clinical results and dislocation rate at a minimum follow-up of 5 years.

## Materials and methods

### Patients

This was a retrospective study performed on patients operated on in 2016 with a total hip arthroplasty (THA) by a single surgeon experienced in hip surgery. The inclusion criteria were the use of a third-generation DMC with a pre-impacted femoral head in a primary total hip replacement for osteoarthritis. Revision surgeries, THA for neck fractures, cemented DMCs, and previous hip infections were excluded. The minimum follow-up required was five years.

### Implants

**Figure 1:**
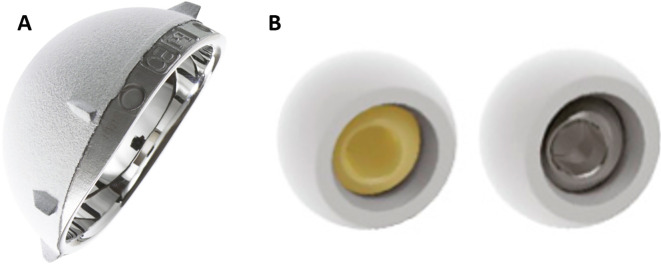
Quattro^®^cup, hemispherical cup with partially extended rim for additional joint stability (a). Pre-impacted head and liner, this solution, in addition to saving operative time, handling and storage, makes it possible to secure the impaction which is done perfectly in the axis in a robotized way and thus protects the third articulation, in particular, the head capture mechanism (b).

The Quattro cementless dual-mobility cup (Lepine^®^, Genay, France) was used in all cases (Figure 1a). It is a third-generation cobalt-chromium alloy acetabular cup with a bilayer coating consisting of a sublayer of pure porous titanium covered by plasma-sprayed hydroxyapatite. The titanium sublayer is obtained by vacuum-plasma spraying, which prevents the interposition of damaging titanium oxides and the weakening of its cohesion with the implant or of the implant itself. The acetabular shell is a half-sphere augmented by a partial cylindrical extension, increasing the coverage angle until 12° allowing additional joint stability. The primary fixation is ensured by eight equatorial fins. The inner surface of the cup is highly polished without any holes for screws or spikes, or additional fixation devices. The polyethylene liner is machined from standard UHMWPE sterilised by ethylene oxide. It is five-eighths of a sphere and features a narrowed section in which the head is secured using a bearing press. In addition, a specific chamfer was made to decrease contact between the retention rim and the neck of the femoral component. The bearing couple was either metal-polyethylene (cobalt-chrome) for 22.2 mm head (46 and 48 mm cups) or ceramic-polyethylene (BIOLOX^®^delta, CeramTec GmbH, Plochingen, Germany) for 28 mm head (cups over 50 mm). All the heads were pre-impacted in the polyethylene in the factory (Figure 1b).

The femoral stem used was collared, cementless with a nonporous fully hydroxyapatite coating on a forged titanium alloy stem, a proximal quadrangular cross-section and an elliptic mirror-polished neck (Targos^®^, Lepine, Genay, France) [23].

### Surgical technique

Patients were operated on either by direct anterior approach (DAA) without any traction table [24] or by mini-invasive posterior approach [25]. In our institution, DAA is standard, except for body mass index (BMI) ≥ 40 kg/m^2^, complex THA requiring corrective osteotomy (e.g., congenital hip dysplasia, corrective leg length discrepancy), over 85 years old with osteoporosis, or previous femoral or pelvic osteotomy [26, 27]. DMCs are used for all patients operated by posterior approach and for patients operated by anterior approach if they were older than 65 years of age and if there was a high risk of dislocation (e.g. epilepsy, Parkinson’s disease, extensive arthrolysis, spinal fusion…).

### Data collection

The demographic data (age, gender, BMI, etiologies) and a pre-operative harris hip score (HHS) were collected. Postoperative clinical assessment was evaluated using the HHS and by collecting complications by an independent observer. The results were classified as: poor (HHS < 70), fair (HHS = 70–79), good (HHS = 80–89), and excellent (HHS = 90–100) [28].

On the postoperative pelvic AP views, the measurements of implant positioning were performed by an independent observer. Cup abduction was the angle between the cup axis and the parallel between the inter-teardrop line. The anteversion was calculated using Widmer’s method [29]. The cup positioning was considered in the safe zone if abduction was between 30° and 50° and anteversion between 10° and 30° [30]. The cup overhang was evaluated on a Lequesne profile view.

Our radiological analysis also focused on socket migration and evaluation of the bone-prosthesis interface. Radiolucent lines (>2 mm) or gaps in the different areas described by DeLee and Charnley [31] were also recorded.

### Ethical approval

All procedures performed in studies involving human participants were in accordance with the ethical standards of the institutional and/or national research committees and with the 1964 Helsinki declaration and its later amendments or comparable ethical standards. For this study, formal consent was not required.

### Statistical analysis

Continuous variables were described by their mean value, standard deviation and minimum and maximum values. Qualitative variables were summarized as percentages. The Kaplan-Meyer method was performed to illustrate the survival of implants. The statistical analysis was performed using XLSTAT^™^ software (version 2021, AddInsoft, Paris, France).

## Results

**Figure 2:**
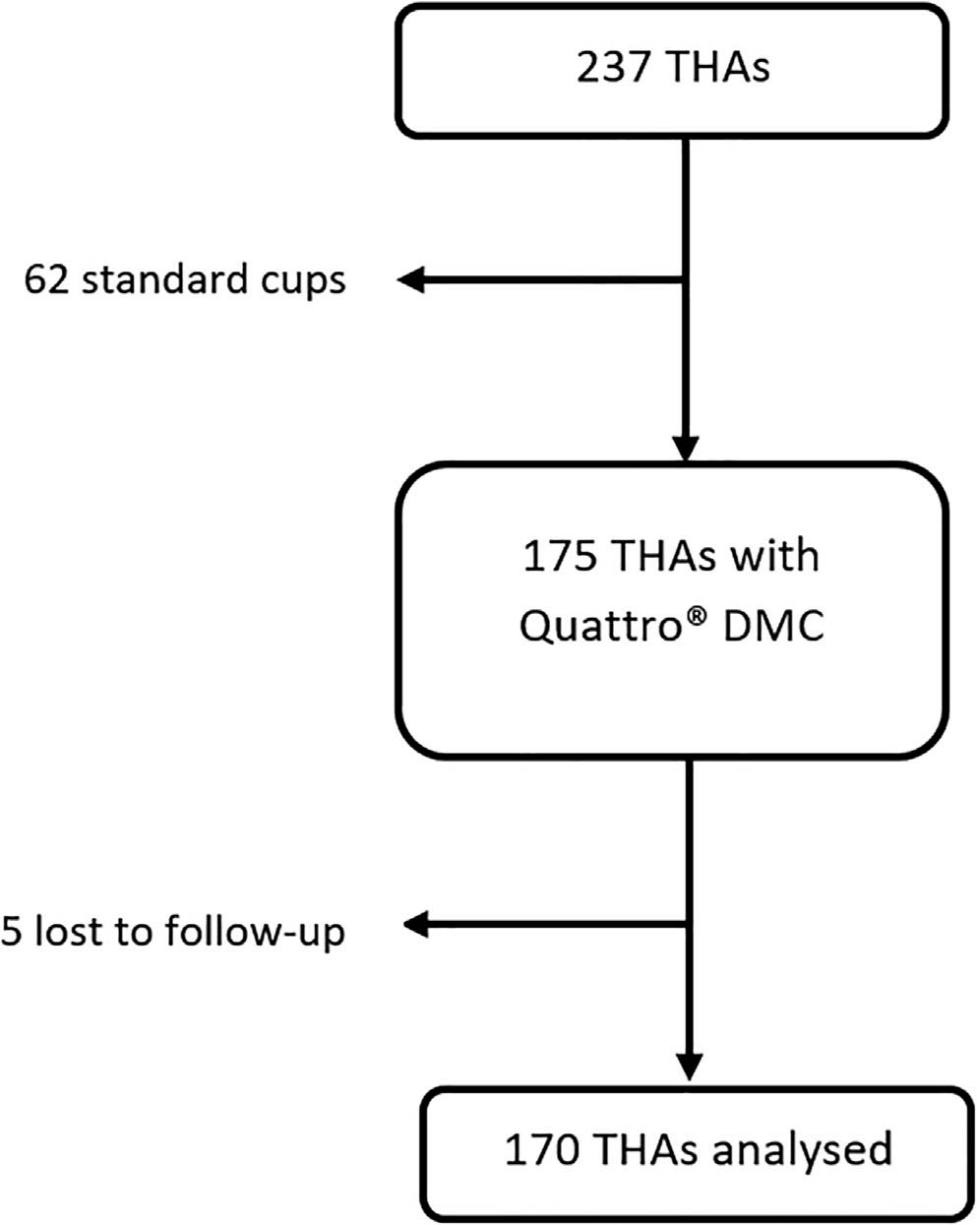
Flowchart, THA: total hip arthroplasty, DMC: dual mobility cup.

Two hundred and twenty-seven THAs were performed in our institution in 2016. Sixty-two hips (26%, 62/237) were excluded as they received a standard cup. One hundred and seventy-five hips (74%, 175/237) (167 patients) met the inclusion criteria. Five hips (2.9%, 5/175) were lost to follow-up and excluded from the postoperative analysis (Figure 2). The mean follow-up period was 70 ± 2.9 months [63.6–77]. Ninety-eight hips (58%, 98/170) were operated by DAA, and 72 (42%, 72/170) by posterior approach. Demographic data and per-operative data are presented in Table 1.

**Table 1:** Pre-operative demographic data and per-operative data.

Parameters	Patients (*n* = 170)
Pre-operative demographic data	
Gender (%F)	120 (70.6%)
Mean age (years)	72.2 ± 8.0 [31.8–93.0]
Age (years)	
<65	30 (17.6%)
[65–75]	77 (45.3%)
[75–85]	46 (27%)
≥85	17 (10.1%)
Mean BMI (kg/m^2^)	27.8 ± 4.6 [17.5–48.4]
BMI (kg/m^2^)	
<30	110 (4.7%)
[30–35]	35 (20.6%)
[35–40]	14 (8.2%)
≥40	11 (6.5%)
Etiology	
Primitive	150 (8.2%)
ONFH	9 (5.4%)
Fracture sequelae (cotyle and femoral neck)	8 (4.7%)
DDH	1 (0.6%)
Osteochondromatosis	1 (0.6%)
Osteochondritis	1 (0.6%)
Pre-op HHS	48.3 ± 6.0 [14.0–70.0]
Per-operative data	
Approach	
Anterior	98 (58%)
Posterior	72 (42%)
Acetabular cup size	51.1 ± 2.9 [42–60]
Head size	
22.2 mm (Cobalt-chrome)	48 (28.2%)
28 mm (Ceramic)	122 (71.8%)

BMI = body mass index, ONFH = osteonecrosis of the femoral head, DDH = developmental dysplasia of the hip, HHS = harris hip score.

**Figure 3:**
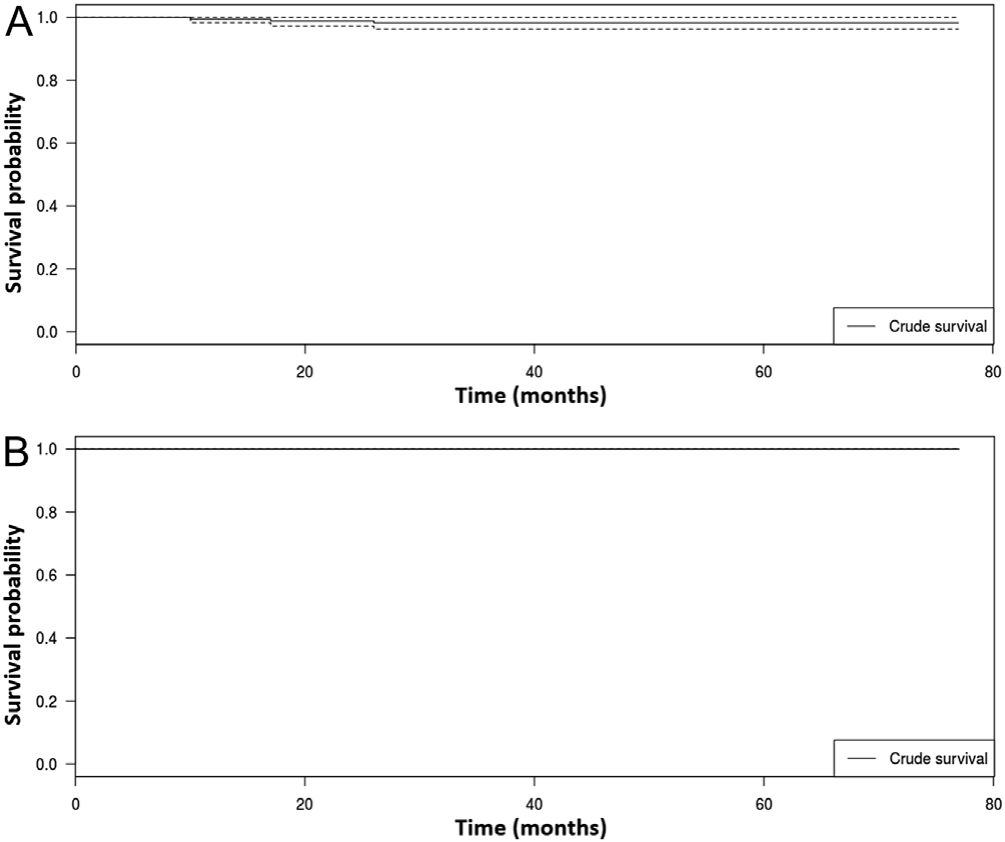
Survival analysis of the acetabular cup. (a) Global and (b) excluding septic etiologies.

There were five infections (2.9%, 5/170): two cases (1.2%, 2/170) of early acute infection treated by DAIR (debridement, antibiotics and implant retention) and three cases (1.7%, 3/170) of chronic sepsis which resulted in one acetabular loosening that underwent a one-step bipolar revision (0.6%, 1/170) at 16.6 months and two chronic pain that underwent bipolar revision (1.2%, 2/170) at 9.7 and 25.9 months. Three other hips needed a revision surgery (1.7%, 3/170): two for aseptic femoral loosening with a unipolar revision (1.2%, 2/170) and one for peri-prosthetic fracture with femoral osteosynthesis (0.6%, 1/170). At 77 months, the global survival probability of the cup was 98.2% ± 1, survival probability of the cup excluding septic aetiology was 100% (Figure 3). There was no aseptic acetabular loosening or postoperative dislocation recorded, nor any iliopsoas-impingement or symptomatic cam-effect (Table 2).

**Table 2:** Intra and postoperative complications, and postoperative functional outcomes at the last follow-up.

	Patients (*n* = 170)
Surgical revision	8 (4.7%)
Including implant removal	5 (2.9%)
cup	3 (1.8%)
femoral stem	2 (1.2%)
Major complication	
Dislocation	0
Intra-prosthetic dislocation	0
Early acute infection–Chronic infection	2 (1.2%)
Acetabular cup loosening	
septic	1 (0.6%)
aseptic	0
Femoral loosening	(1.2%)
septic	0
aseptic	2 (1.2%)
Post-operative peri-prosthetic fracture	1 (0.6%)
Acetabular side	0
Femoral side	1 (0.6%)
Minor complication	
Ilio-psoas impingement	0
Symptomatic came effect	0
Medius gluteus tendinitis	2 (1.2%)
Per-operative femoral fracture	1 (0.6%)
Greater trochanter fractures	3 (1.8%)
Femoral wrong way	0
HHS	96 ± 4.5 [50–100]
Repartition of HHS	
Excellent (≥90)	153 (90%)
Good (80–89)	13 (7.6%)
Fair (70–79)	2 (1.2%)
Poor (<70)	2 (1.2%)

HHS: harris hip score, DAIR = debridement, antibiotics and implant retention.

**Figure 4:**
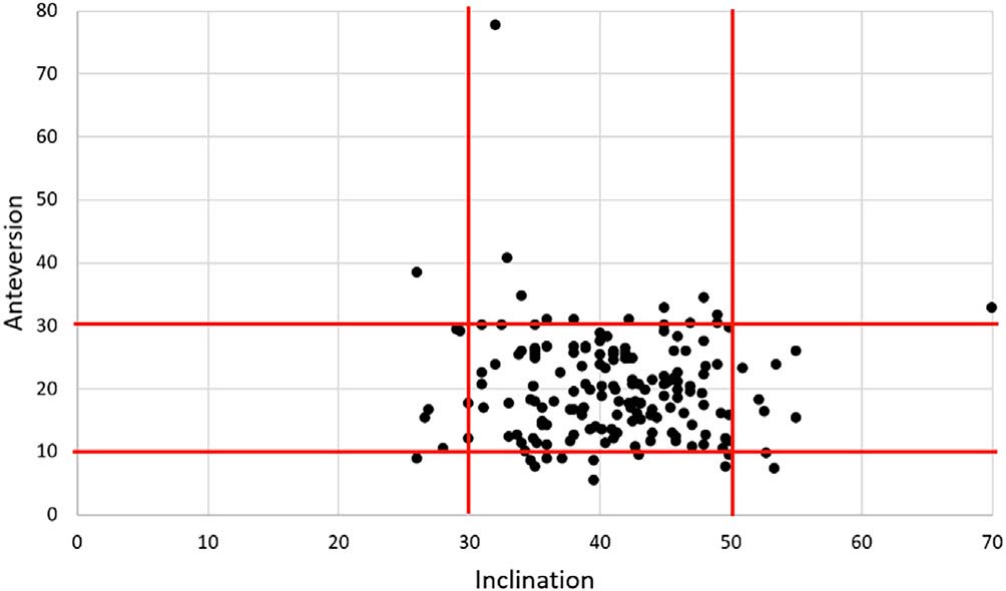
Cup positioning according to safe zones. 90.6% (154/170) of the cups were in the safe zone in the frontal plane, 86.5% (147/170) in the transversal plane, and 80% (136/170) in both planes.

**Figure 5:**
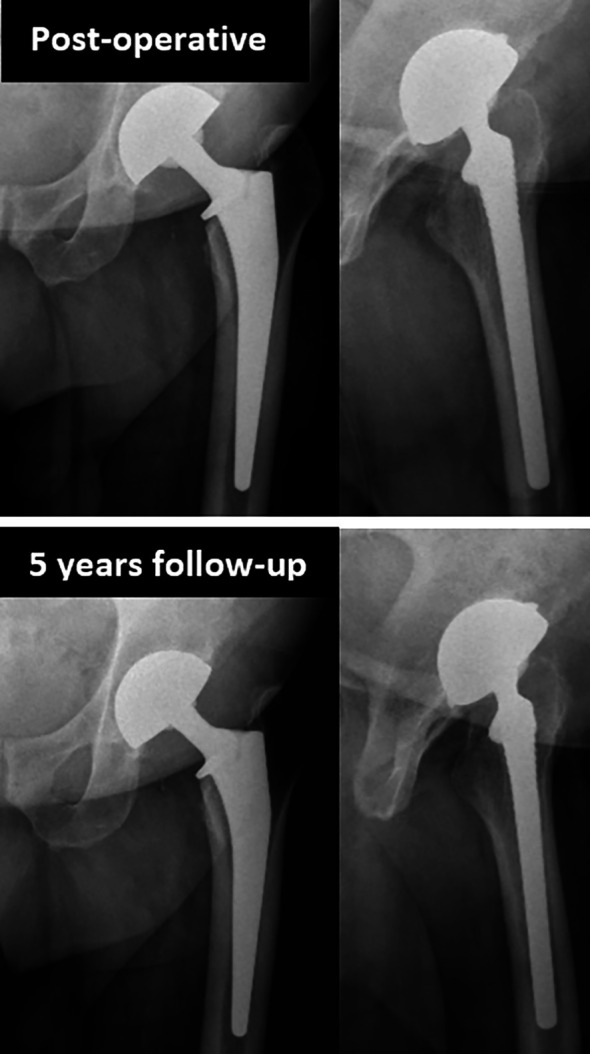
No eccentric position of the femoral head in the cup (as a sign of polyethylene wear) was found at the last follow-up.

There was a significant improvement of the HHS from pre-operatively (48.3 ± 6.0 [14.0–70.0]) to post-operatively (96 ± 4.5 [50–100]) (*p* < 0.0001) (Tables 1 and 2). 90% (153/170) sustained excellent results with HHS ≥ 90 (Table 2). 90.6% (154/170) of the cups were in the safe zone in the frontal plane, 86.5% (147/170) in the transversal plane and 80% (136/170) in both planes (Figure 4). The mean inclination was 41.0 ± 5.3 [26.0–70.0], mean anteversion was 19.8 ± 6.3 [0–77.8]. Two patients had an anterior cup overhang (1.2%, 2/170) of 4 and 1 mm, with no associated iliopsoas syndrome. There was no evidence of cup migration, nor any radiolucent lines (signs of loosening), or any eccentric position of the head within the cup noted (Figure 5).

## Discussion

The main finding of this study is that the mid-term survivorship of this modern dual mobility cup with a pre-impacted femoral head was excellent in this study (98.2% at 6.4 years). No dislocations were recorded, nor any specific complications classically described for dual mobility cups (psoas impingement, IPD, symptomatic cam effect) were seen at 70 months FU. The survival rate excluding septic complications was 100% at 6.4 years.

The first-generation DMCs, which did not have hydroxyapatite coating to promote osteointegration, were associated with an increased risk of aseptic loosening and a higher occurrence of radiolucent lines [9, 32]. Second generations of DMCs with a double coating of titanium and hydroxyapatite allow for a theoretically better osteointegration leading to a theoretically higher survival probability. Even though direct statistical comparisons are not possible, survivorship seems higher with second-generation DMC with a survival probability >97% at 5–11 years in the literature [16–18, 33–41] and in our study. The large sliding surface of DMCs is often pointed out as a source of particle release, yet an in-vitro study found with a UHMWPE that the wear performance between a DMC and a standard cup with a 28 mm ceramic head was equivalent [42]. Even though last-generation polyethylene is not already the reference in DMC, some authors recommend their use in daily practice based on finite element analysis, even obtaining with a 22.2 mm femoral head a similar amount of volumetric wear than a conventional acetabular component with a 32-mm-diameter femoral head against XLPE [22, 43]. If we consider such excellent results, we might expect that new generations of DMC enhanced by the latest surface coating (highly porous titanium) [44] and sliding surface (cross-linked polyethylene(XLPE)) [45] technologies continue to ensure optimal survival at the longest follow-up.

Optimal stability is the aim for dual mobility use in primary and revision hip replacement. There were no cases of dislocation in our series, which corroborate the literature [6, 16–18, 33–40]. These results are all the more relevant as there was a high proportion of the population which was at high risk of dislocation (36.9% of obese patients, 38.6% ≥75 years old) [46]. This protective effect on obese patients has already been described [47], as well as in elderly patients [48].

The partially extended rim of this implant allows an increased coverage angle of up to 12° participating in this anti-dislocation shield [49]. This increased coverage being only on the upper side, this increased stability was not at the expense of a rising risk of psoas impingement nor to symptomatic cam effect as those complications were not recorded in this study. Other designs can be used to face this compromise, and some manufacturers use a cylinder-spheric design (sphere augmented by a cylinder of 3 mm at the equatorial area) [38], while others use an anatomical design (anterior notch to accommodate the iliospsoas tendon, and a posterior and inferior prominence to increase the congruence) [45]. Whatever the design, the surgical technique is crucial, and appropriate cup positioning with no anterior impingement is mandatory.

Not recorded as well in this study, IPDs seem to become anecdotal. None of the recent publications following new-generation DMC found any intraprostatic dislocations (IPDs) [6]. The source of this improvement is multifactorial. It is mainly due to the improvement of the quality of the polyethylene and the improvement of the 3rd articulation (chamfer, thin polished round neck) [42]. The originality of the cup used here is that it respects the third articulation as much as possible by standardizing the impaction of the heads robotically prior to surgery in order to avoid any damage to the head capture mechanism. Even if at mid-term follow-up it is difficult to prove that the benefit of this particular construct is not only theoretical, the current study does support its safety.

Particular care has been taken to use ceramic heads as frequently as possible as they are known to give less fretting corrosion at the head–neck interface (trunionitis) [50]. By analogy with the metal on metal bearing surfaces used in the past, we also know that it is preferable to avoid metal on metal head-neck interface as much as possible when placing large-diameter heads since these are associated with an increased risk of trunionitis due to larger frictional torques and larger bending moments are generated on the femoral trunnion [51].

Even though the implementation of a monobloc DMC is known to be more difficult due to the lack of a solid anchorage point at the bottom of the cup for the holder, this study found an excellent global placement of the cup (90.1% well-positioned in the frontal plane, 86% in the transversal plane). Those results are similar to those found with standard cups [28, 30], keeping in mind that 42% of the procedures were performed through DAA with fluoroscopic control, which is known to allow for good control of the cup positioning [52].

There are some limitations to this study that need to be acknowledged. The assessment of implant position was on radiographs and not on CT scan, however, a CT scan is not recommended for routine THA follow-up. This would have exposed study patients to increased radiation and would not reflect common practice. Another limitation is the mid-term follow-up, knowing that the ideal follow-up to study loosening would be after 10–15 years, but this is the first report of the pre-impacted femoral head in modern DMC and considering the mean age of 72 years in our series, the mean FU of 6 years is relevant.

This study has several advantages, including a large cohort size with almost no loss at follow-up of a minimal 5y with the same implant and the same surgeon. Our results at mid-term FU seem to support the routine use of DMC in primary hip replacement for the patient at risk of instability since the stability is achieved without specific complications related to the implant.

## Conclusion

This study showed an excellent survival of this modern dual mobility cup at a mid-term follow-up. No dislocations were recorded, nor any specific complications (intraprosthetic dislocation, psoas impingement, symptomatic cam effect), which are feared with dual mobility cups were seen. The authors would recommend using dual mobility implants for primary hip replacement in patients at risk of instability.

## Data Availability

Not applicable.

## Design

A retrospective observational study, level IV.
